# The Effects of Shellac and Glycerol on the Physicochemical Properties of Chitosan Films

**DOI:** 10.3390/polym17101298

**Published:** 2025-05-09

**Authors:** Patrycja Brudzyńska, Alina Sionkowska

**Affiliations:** Laboratory for Biomaterials and Cosmetics, Faculty of Chemistry, Nicolaus Copernicus University in Torun, Gagarina 7, 87-100 Torun, Poland

**Keywords:** chitosan, shellac, glycerol, biopolymers, polymeric films

## Abstract

Chitosan has been investigated for applications in biomaterials, pharmaceuticals, food, biodegradable packaging, and adsorbents. Various natural substances have been incorporated to modify chitosan properties and fabricate functionalized materials. Shellac, a lac-based biopolymer, is a biodegradable, edible, and biocompatible compound used in the food and pharmaceutical industry. Several materials combining chitosan and shellac were studied for packaging, food preservation, or drug delivery systems. In the present study, chitosan films enriched with shellac and glycerol were prepared. The physicochemical characterization of biopolymeric materials was performed (atomic force microscopy, scanning electron microscopy, contact angle and surface free energy, attenuated total reflectance Fourier-transform infrared spectroscopy, thermogravimetric analysis, mechanical testing, and swelling analysis). The effect of shellac and glycerol on chitosan materials was investigated. As a result, modified chitosan films were homogeneous and were characterized by increased elongation at break, surface free energy, and surface hydrophilicity (samples containing higher concentration of shellac), as well as decreased tensile strength, swelling degree determined at a pH of 7.4, and surface roughness in comparison to pure chitosan films. No significant differences in the thermal properties of modified chitosan-based materials were observed. The incorporation of shellac and glycerol influenced the physicochemical properties of chitosan films, which may act as a matrix for incorporating active substances for use in biomaterials, food packaging, cosmetics, or pharmaceuticals.

## 1. Introduction

Biopolymer-based materials are broadly fabricated and investigated for the development of novel wound healing materials, regenerative biomaterials, drug delivery systems, functional food, biodegradable food packaging, cosmetics, or adsorbents for contaminant removal. Chitosan is widely implemented and evaluated in these distinguished applications [1,2,3,4,5,6,7,8,9,10]. This biological macromolecule is produced by the deacetylation of chitin, extracted mainly from the exoskeletons of crustaceans, and consists of -(1,4)-bonded D-glucosamine and N-acetyl-D-glucosamine units. In chitosan molecules, a minimum of 60% deacylated units are included [11]. Chitin, together with cellulose, is one of the most abundant polysaccharides [12]. Molecular weight and the degree of deacetylation are key features characterizing this polycationic polymer, strictly related to its solubility. This linear chitin derivative is characterized by biocompatibility, non-toxicity, muco-adhesiveness, wound healing, antioxidant, and antimicrobial properties [12,13]. The latter were demonstrated against fungi and Gram-positive and Gram-negative bacteria; however, the influence of factors such as viscosity, solubility, and surface charge as well as environmental factors like humidity and light on antibacterial activity are still not fully recognized [12,14]. The presence of amino (-NH_2_) and hydroxyl (-OH) functional groups in chitosan enables its chemical modification [15,16]. Chitosan derivatives, namely carboxymethyl chitosan, hydroxyethyl chitosan, N-succinyl chitosan, quaternized chitosan, chitosan phosphate, and carboxymethyl methacrylate chitosan, are also of great interest and are implemented in a broad range of applications, for instance, as an antibacterial agent, in bone tissue engineering, for drug administration, as well as for polypeptide or gene delivery [16,17]. To modify the physicochemical and biological properties of chitosan in order to develop functionalized materials in different forms, for example, films, hydrogels, scaffolds, and many others, meeting the present requirements, multiple substances of natural origin are incorporated [18,19,20].

Shellac, a natural material of animal origin, is a lac-based product obtained from the insect secretion, mainly *Kerria lacca*, which can be found on trees growing in India and Thailand. This hard, brittle, amorphous solid is a low-molecular-weight complex compound consisting mainly of polyester resin with a small content of dyes and waxes [21,22]. Shellac, an amphiphilic compound, is a type of resin consisting of a long polyester chain with inter- and intra-esters, polyhydroxy carboxylic acids responsible for its hydrophilicity, such as sesquiterpene acids, for instance, jalaric acid, which constitutes 25% of shellac components, and aliphatic hydroxy acids that ensure hydrophobicity, like aleuritic acid, which constitutes 35% of shellac components. It is a regularly oriented semi-crystalline biopolymer, which is characterized by low density [23]. Above the glass transition temperature, equal to 40–50 °C, shellac transforms to a soft and thermoplastic material, while at a temperature of 90 °C, it melts. By lowering the temperature, the crystallization of shellac decreases [23]. Shellac undergoes aging and is prone to polymerization and self-esterification. This lac-based material is insoluble in water but dissolves in ethanol, acetone, and alkaline solutions [21]. It is a biodegradable, edible, non-toxic, biocompatible, and cell-compatible biopolymer, which can be used in tissue engineering. Shellac found applications in the food and pharmaceutical industry, but also in paints, varnishes, polishes, adhesives, and textile dyes. In cosmetic formulations, shellac can act as a binder [21]. Furthermore, this raw material can be used in the encapsulation process, for example, in perfumes [21,24].

Several materials consisting of chitosan and shellac were fabricated and investigated for various applications. For food packaging applications, Yuan et al. [25] prepared shellac nanoparticles, which were subsequently incorporated into chitosan films. Moreover, chitosan films with curcumin-loaded shellac nanoparticles were also prepared [25]. Chitosan and shellac emulsion-based coatings enriched with pine needle essential oil were designed as packaging characterized by a uniform texture, suitable water vapor, oxygen, and ultraviolet barrier properties, as well as a bacteriostatic effect [26]. Ramasamy et al. [27] prepared and evaluated a biocomposite fabric made of chitosan and shellac, indicating appropriate thermal, mechanical, and barrier stability for application in sustainable packaging [27]. Moreover, chitosan/shellac composite membranes with ZnAl_2_O_4_ and CuAl_2_O_4_ nanoparticles were fabricated as promising novel materials with antibacterial activity for food packaging [28]. The chitosan and shellac combination was also involved in the development of bio-based adhesives for potential application in packaging papers [23]. For food preservation, Ma et al. [29] developed a chitosan film with porous shellac hydrogel microparticles, ensuring enhanced carbon dioxide, oxygen, and water vapor permeability as well as suitable antifogging properties, degradability, and optical transparency [29]. For the same purposes, bilayer films obtained from polyvinyl alcohol and shellac with carboxymethyl chitosan and copper oxide nanoparticles with antimicrobial activity were designed [30]. Multiple delivery systems for drugs and bioactive substances were constructed, including biopolymers such as chitosan and shellac [31,32,33,34,35,36,37,38,39]. Emulsion gels with shellac nanoparticles and chitosan complexes, serving as emulsifiers and gelling agents, were constructed as a β-carotene delivery system, enabling the appropriate retention and bioaccessibility of selected carotenoids [31]. Biocompatible nanoparticles were prepared with shellac and quaternized chitosan for nutraceutical quercetagetin encapsulation with suitable storage stability, antioxidant activity, and controlled release [32]. Liu et al. [33] constructed nanoparticles made of zein and shellac coated with chitosan and loaded with quercetin, which displayed environmental stability and enhanced antioxidant activity with controlled release in the simulated gastrointestinal solutions [33]. Chitosan and shellac nanoparticles obtained by ionic crosslinking were used as a protein delivery system, and bovine serum albumin was encapsulated [34]. The alginate–shellac gel particles coated with chitosan were fabricated by Zhu et al. [35] as a potential oral delivery system for superoxide dismutase [35]. Shellac and chitosan were also combined for the ciprofloxacin drug delivery system in the form of nanocarriers, improving its antibacterial and anticancer activity. Nanoparticles exhibited low cytotoxicity, a high inhibition rate for melanoma cells, a low inhibition rate for normal cells (HFF), and were effective antibacterial agents for positively and negatively charged bacteria [36]. The oral delivery system of cefaclor based on chitosan–alginate beads coated with shellac was designed, and the suitable retardation of drug release and significant antimicrobial properties were observed [37,40]. An enteric-coated tablet for the oral delivery of ceftriaxone, a drug with antibacterial activity, was constructed with the use of chitosan, while an enteric coat of the core was prepared from shellac and glycerol tristearate. The proposed system enhanced drug bioavailability and could be a promising solution for drug delivery [38,40]. Nanoparticles consisting of chitosan, shellac, and a selected drug such as meclizine hydrochloride were fabricated by ionic crosslinking and included into the core in coated tablets to prepare dual-function pharmaceuticals [39,40].

In the present study, chitosan films incorporated with various amounts of shellac and glycerol as a plasticizer were prepared. The physicochemical characterization of biopolymer-based materials was performed. To investigate the chemical structure, the attenuated total reflectance Fourier-transform infrared (ATR-FTIR) spectra were registered. Films’ surfaces were characterized by atomic force microscopy (AFM), scanning electron microscopy (SEM), as well as the contact angle. Surface free energy was also calculated. Thermal properties were examined by thermogravimetric (TGA) analysis. The effects of shellac and glycerol on the mechanical properties and swelling degree of chitosan films were evaluated. Several reports concern the chitosan and shellac combination, while the properties of both biopolymers enable their use in biomaterials and other biomedical purposes. Separately, chitosan and shellac have been successfully used in those fields, for example, for wound healing or drug delivery systems. To the best of our knowledge, chitosan films incorporated with shellac and glycerol have not been extensively and fully investigated for biomedical applications. Our research focused on the preliminary physicochemical characterization of materials, which is essential in the initial stage of biomaterial design and their further fabrication.

## 2. Materials and Methods

### 2.1. The Film Preparation

**Figure 1:**
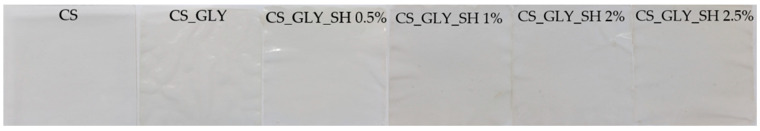
Photos of chitosan-based films. From the left: chitosan film, chitosan film with glycerol addition, and chitosan films with glycerol and shellac addition of 0.5, 1, 2, and 2.5%, respectively.

Low-molecular-weight chitosan (Sigma Aldrich, St. Louis, MO, USA) was dissolved in a 0.1 Mol/L acetic acid (Stanlab, Lublin, Poland) solution to obtain a 2% chitosan solution. Shellac (Pol-Aura, Zawroty, Poland) was dissolved in ethyl alcohol (Chempur, Piekary Śląskie, Poland) to prepare a 5% solution. To each sample containing 25 g of chitosan solution (CS), 0.25 g of glycerol (GLY) (Chempur, Piekary Śląskie, Poland) was added, and, subsequently, an appropriate amount of shellac solution (SH) was added. Based on the shellac-to-chitosan ratio, four samples (0.5%, 1%, 2%, 2.5%) were prepared. The individual component contents in each film are summarized in Table 1. Biopolymer films were obtained by the solution casting method (the solution was poured on a 10 × 10 cm polystyrene plate) at a temperature of 37 °C. The thickness of all prepared films was similar and equal to about 0.04 mm. Samples were marked as follows: CS, CS_GLY, CS_GLY_SH 0.5%, CS_GLY_SH 1%, CS_GLY_SH 2%, and CS_GLY_SH 2.5%. Photos of the prepared films are presented in Figure 1.

### 2.2. ATR-FTIR Spectroscopy

To register the IR spectra of film samples, a Nicolet iS10 spectrometer equipped with a diamond ATR accessory (Thermo Fisher Scientific, Waltham, MA, USA) was used (resolution 4 cm^−1^, 64 scans, wavenumber range from 400 to 4000 cm^−1^).

### 2.3. SEM Analysis

Film samples were imaged by a SEM (LEO Electron Microscopy Ltd., Cambridge, UK). Samples were covered with a gold layer before analysis. Images were recorded at 1000× magnification.

### 2.4. AFM Analysis

The AFM technique with tapping mode at room temperature (MultiMode Scanning Probe Microscope Nanoscope IIIa; Digital Instruments Veeco Metrology Group, Santa Barbara, CA, USA) was used to investigate the surface morphology and roughness of chitosan films with the shellac and glycerol addition. Nanoscope software (v6.11) (Bruker Optik, Ettlingen, Germany) was applied to calculate the roughness parameters for the scanned area 5 μm × 5 μm.

### 2.5. Contact Angle Measurements and Surface Free Energy Determination

The contact angle was measured by a goniometer with a drop shape analyzer system (DSA 10, Krüss, Germany). For each film, about 10 measurements were performed for two liquids: a hydrophobic liquid, diiodomethane (D), and a hydrophilic liquid, glycerol (G). Analyses were conducted at room temperature. The surface free energy was calculated by the Owen–Wendt method.

### 2.6. TGA

A thermal analyzer Jupiter STA 449 F5 (Netzsch, Selb, Germany), combined with a spectrometer FTIR Vertex 70V (Bruker Optik, Ettlingen, Germany), was used to study the thermogravimetric behavior of the film samples at the temperature range from 20 to 600 °C at a heating rate of 20 °C/min in the nitrogen atmosphere.

### 2.7. Mechanical Testing

The mechanical properties of biopolymer films such as Young’s Modulus (GPa), elongation at break (%), and tensile strength (MPa) were evaluated by a mechanical testing machine (Z.05, Zwick and Roell, Ulm, Germany) with the speed starting position of 50 mm/min, the speed of the initial force of 5 mm/min, and the initial force of 0.1 MPa. Data were collected with the TestXpert II 2017 program. A one-way ANOVA test was used to indicate statistically significant differences between film samples.

### 2.8. Swelling Analysis

For prepared chitosan-based materials, the swelling degree was investigated. Five square-shaped samples with a weight of about 0.0050 g were prepared for each film. Before analysis, samples were dried in a vacuum dryer at a temperature of 45 °C. Subsequently, samples were placed in phosphate-buffered saline (PBS; Life Technologies Limited, Paisley, Scotland, UK) at a temperature of 37 °C and were weighed after 0.5 h, 1 h, 2 h, 4 h, 8 h, 24 h, 48 h, and 72 h. The following equation was used to calculate the swelling degree:

$$swelling=\frac{\left(m_{t}-m_{0}\right)}{m_{0}}\times 100\%\;\;[\%]$$

*m_t_*—weight of the material after immersion in PBS [g].

*m*_0_—initial weight of the material [g].

## 3. Results

### 3.1. ATR-FTIR Spectroscopy

**Figure 2:**
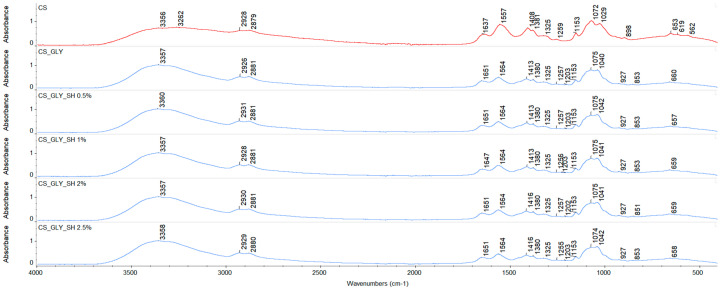
FTIR spectra for chitosan films with glycerol and shellac addition (experiment conditions: resolution = 4 cm^−1^; wavenumber range = 400–4000 cm^−1^; 64 scans).

**Figure 3:**
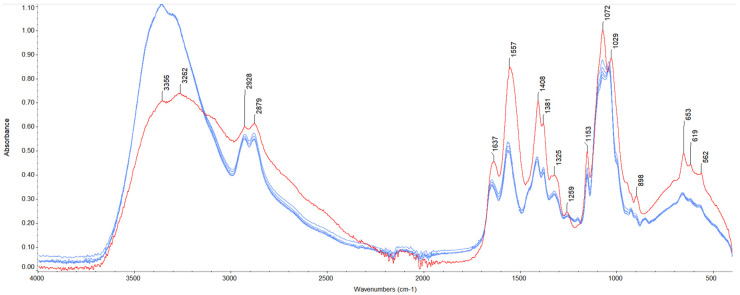
FTIR spectra for chitosan films with glycerol and shellac addition (experiment conditions: resolution = 4 cm^−1^; wavenumber range = 400–4000 cm^−1^; 64 scans).

The spectra of pure chitosan and modified samples are depicted in Figure 2 and Figure 3. FTIR analysis confirmed the presence of functional groups, and characteristic bands for chitosan were found on the spectra, which are summarized in Table 2. For modified samples, alterations of the bands to higher wavenumbers were observed for the amide I band, amide II band, bend of -CH_2_ bands, and symmetrical stretch of C-O-C bands. Furthermore, an additional band at 1203 cm^−1^ was noticed, which might be related to the glycerol presence in the modified samples [41]. These slight shifts might be associated with the formation of new interactions between film components.

### 3.2. SEM Analysis

**Figure 4:**
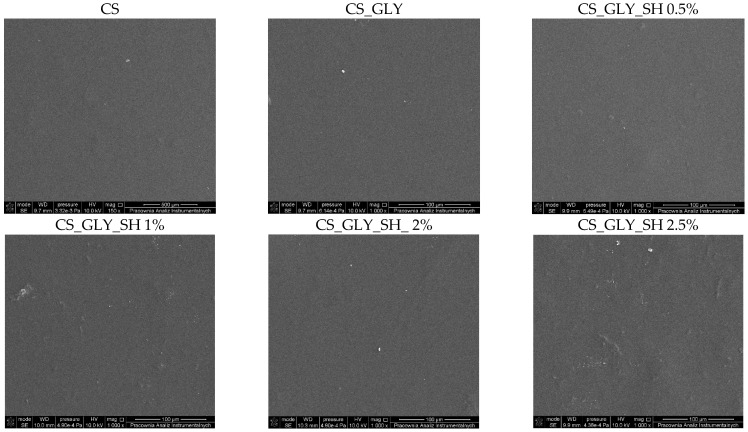
SEM images (at the magnification of 1000×); from the left: chitosan film, chitosan film with glycerol, and chitosan films with glycerol and shellac addition (0.5, 1, 2, and 2.5%, respectively).

The magnified images of the chitosan films, enabling morphology investigation, were produced by SEM. The SEM images of the biopolymer materials are presented in Figure 4. Prepared chitosan films with shellac and glycerol addition displayed a smooth, flat, uniform, and continuous surface without any pores or cracks; however, in comparison to pure chitosan film, a slight undulation of modified films was observed. Shellac and glycerol distribution were homogeneous in the chitosan matrix.

### 3.3. AFM Analysis

**Figure 5:**
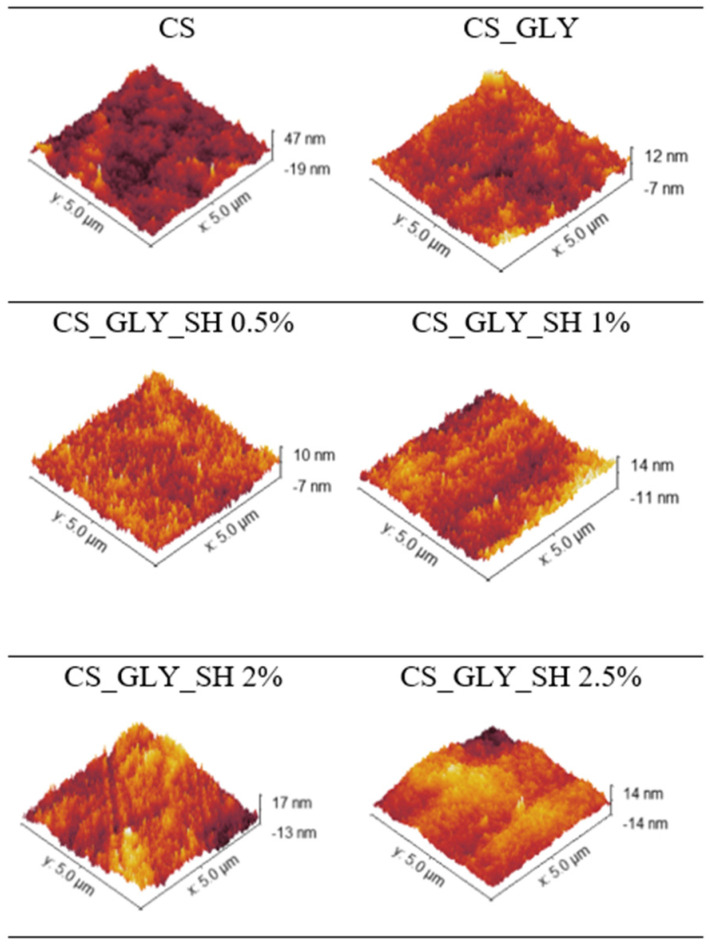
AFM images of the surface of chitosan-based films.

The surface morphology of chitosan-based films was also investigated by AFM. In Table 3, the roughness values of the prepared samples for the scanned area of 5 μm × 5 μm are presented, while in Figure 5, the AFM images are shown. The highest values of the roughness parameters were observed for pure chitosan film, while the glycerol addition caused a decrease in Rq and Ra values. The roughness of the materials with the glycerol and shellac solution addition was related to the concentration of the incorporated biopolymer. Sample CS_GLY_SH 0.5% indicated the lowest values of the roughness parameters among all the samples, whereas 1, 2, and 2.5% of shellac addition caused an increase in the Rq and Ra values in comparison to the chitosan/glycerol film. As shown in the AFM results, slight differences in the roughness parameters Rq and Ra of the prepared films occurred, which means that it can be concluded that glycerol and shellac modified the surface morphology of chitosan films.

### 3.4. Contact Angle Measurements and Surface Free Energy Determination

The results of the contact angle measurement are summarized in Table 4. According to the contact angle results for hydrophilic liquid (glycerol), it can be deduced that 2 and 2.5% of the shellac addition to the chitosan matrix caused a decrease in contact angle values; thus, the surfaces of the investigated samples were characterized by a more hydrophilic nature and greater wettability. This corresponds with the result of the polar component of surface free energy. As can be seen, the incorporation of selected additives contributed to the increase in the surface free energy and, among all the samples, the chitosan film with glycerol and the 0.5% addition of shellac presented the highest values for this parameter.

### 3.5. TGA

**Figure 6:**
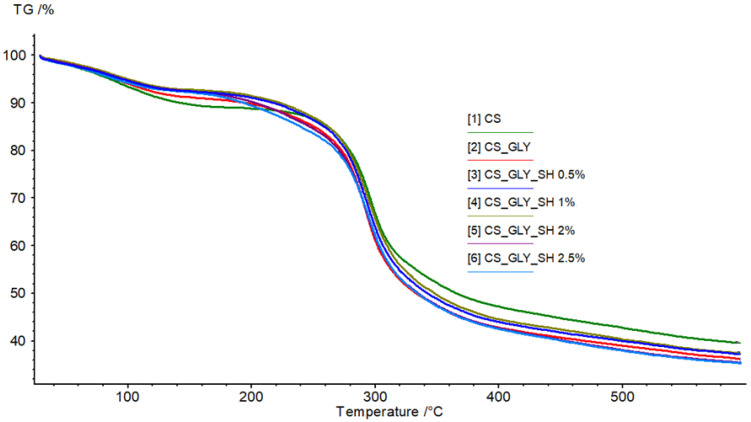
TG curve obtained for unmodified and modified chitosan films (temperature range from 20 to 600 °C at a heating rate of 20 °C/min in a nitrogen atmosphere).

**Figure 7:**
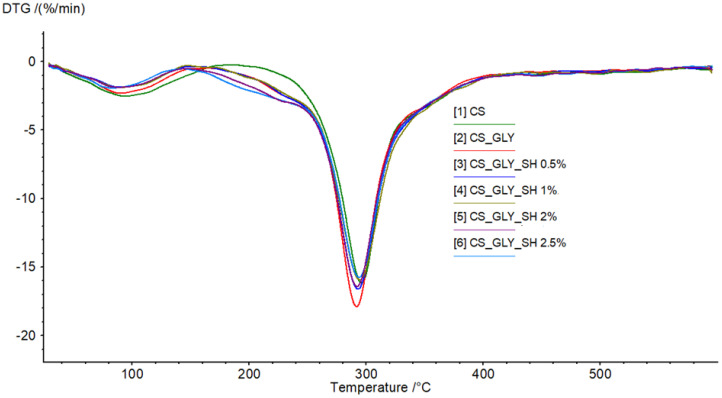
DTG curve obtained for unmodified and modified chitosan films (temperature range from 20 to 600 °C at a heating rate of 20 °C/min in a nitrogen atmosphere).

The thermal properties of chitosan samples were investigated by the thermogravimetry method. The thermal gravimetry (TG) and differential thermal gravimetry (DTG) curves of chitosan-based films are shown in Figure 6 and Figure 7, respectively, while TG and DTG curve parameters are gathered in Table 5. According to thermograms, two stages were distinguished during the thermal decomposition of the samples. The first region probably referred to the elimination of water molecules or solvent residues from the films, and the second one referred to material decomposition. There were no significant differences in thermograms for all samples. In the initial stage, weight loss was about 5%, while in the second stage, it was about 30%. The T_max_ values in both steps were similar and equal to about 90 °C and 290 °C, respectively. In the DTG curve, one peak in the second region of the degradation step may suggest that all the modified samples were homogeneous. Shellac did not influence the thermal stability of the chitosan film significantly; however, as can be seen on the TG curve in the range from about 90 °C to 180 °C, the weight loss and therefore thermal degradation were noticeably greater for the pure chitosan sample and chitosan/glycerol sample.

### 3.6. Mechanical Testing

**Figure 8:**
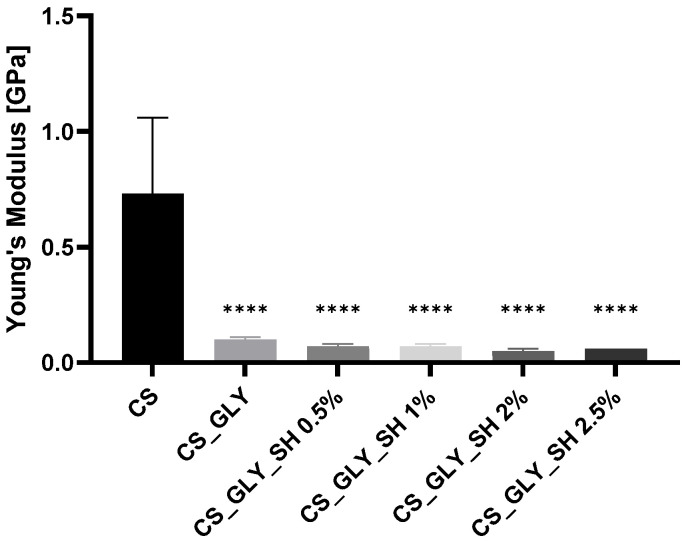
Young’s Modulus for chitosan-based films (statistically significant differences vs. CS sample are indicated as **** *p* < 0.0001).

**Figure 9:**
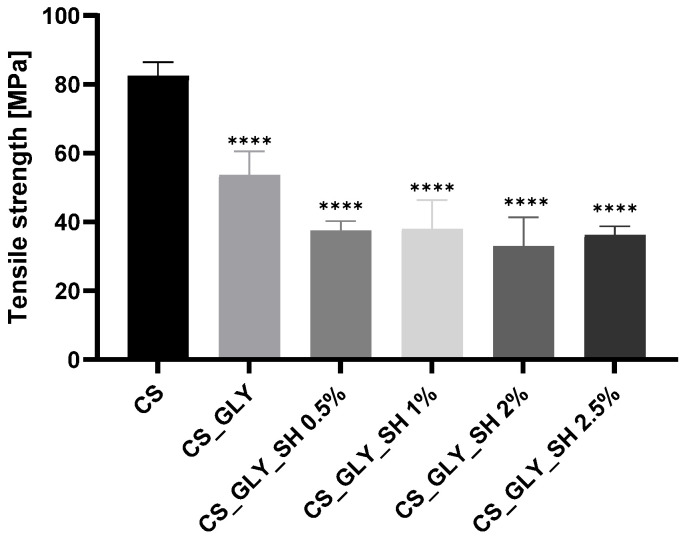
Tensile strength for chitosan-based films (statistically significant differences vs. CS sample are indicated as **** *p* < 0.0001).

**Figure 10:**
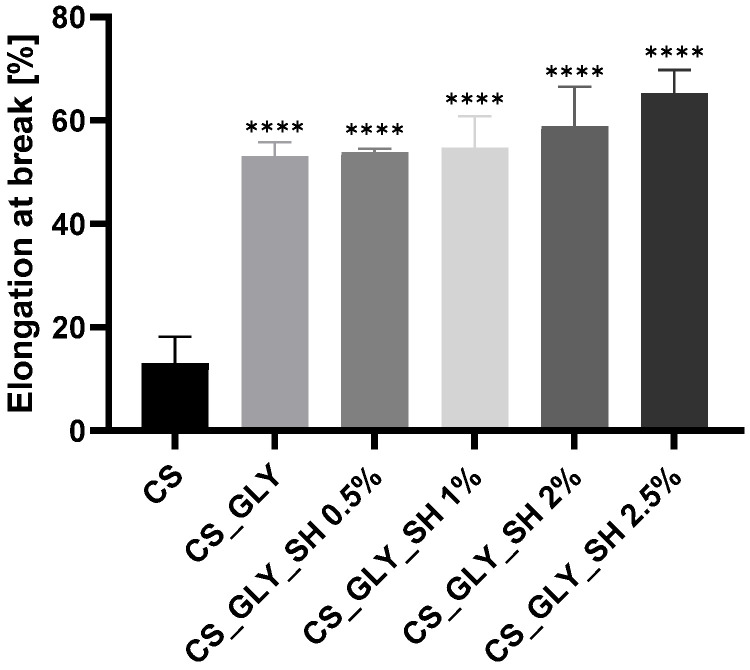
Elongation at break for chitosan-based films (statistically significant differences vs. CS sample are indicated as **** *p* < 0.0001).

According to mechanical testing, the shellac and glycerol addition influenced the mechanical parameters of the chitosan-based materials. Young’s Modulus, tensile strength, and elongation at break parameters for the investigated samples are presented in Figure 8, Figure 9 and Figure 10. For modified samples, significantly lower Young’s Modulus values were observed, which were associated with the glycerol addition. Films enriched additionally with shellac displayed similar values. Tensile strength was also lower for modified films, and the glycerol addition caused a decrease in this parameter, but samples with glycerol and shellac indicated even lower values. However, the concentration of this biopolymer was not determinative. The increase in the values of elongation at the break parameter characterized the modified films; particularly, the sample with the highest concentration of shellac (2.5%) indicated a significantly higher elongation at break and was the most flexible among all films.

### 3.7. Swelling Analysis

**Figure 11:**
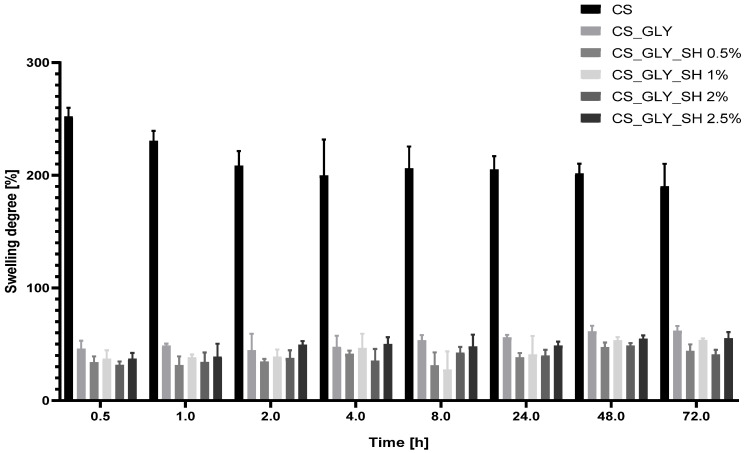
Swelling degree for chitosan-based films.

Swelling degree results for chitosan films are presented in Figure 11. Samples were analyzed in phosphate-buffered saline at a pH of 7.4 for three days. Pure chitosan film was characterized by the highest swelling degree among all samples, which was about 250%, while the additives caused a significant decrease in the films’ swelling degree. Samples enriched with shellac indicated more reduced values than the chitosan/glycerol film. However, the values of the swelling degree for all glycerol and shellac-enriched films were similar, and the influence of shellac concentration was not observed. During three days of swelling analysis, chitosan film disintegration was observed, while modified samples were more stable at a pH of 7.4.

## 4. Discussion

Based on the scientific literature, the fabrication and development of biopolymer materials from natural resources that can find application in many industrial sectors meet the current expectations. Our results contribute to extending the knowledge about chitosan/shellac/glycerol combinations, which are eagerly used for food, packaging, biomedical, or cosmetic purposes. In the context of an investigation of chitosan/shellac materials, the number of studies is growing but still remains limited.

Chitosan was mixed with shellac in several studies. Yuan et al. [25] constructed shellac nanoparticles, which were subsequently incorporated into chitosan films. FTIR analysis corresponds with our results; thus, characteristic bands arising from -OH and -NH stretching vibrations, C=O stretching vibrations, and -NH_2_ bending vibrations were also registered. However, on the spectra of shellac/glycerol-modified chitosan films, we did not observe a peak at 1712 cm^−1^ ascribed to the C=O stretching of carbonyl groups in shellac molecules, which in Yuan et al.’s [25] study increased with an increasing concentration of shellac nanoparticles. This might be associated with a low concentration of shellac incorporated into our chitosan matrices. As no additional bands were found in the FTIR spectra of the modified samples, it was concluded that no chemical bonds were created between biopolymers, and based on the cationic groups present in chitosan and the anionic groups present in shellac, the electrostatic interactions were predicted. Chitosan film enriched with shellac nanoparticles also exhibited improved thermal properties, and with increasing shellac content, a decrease in mass loss occurred; however, three degradation steps were registered, and the second one reflected glycerol evaporation. The smaller weight loss of the modified samples in the first region, based on the TG and DTG curves, could refer to the presence of the hydrophobic groups included in the shellac molecules, contributing to lower water molecule absorption. Based on our thermogravimetric analysis results in a range of 90–180 °C, we also observed slightly less weight loss and thus a decrease in the thermal degradation for shellac-enriched films. Chitosan films with shellac nanoparticles also displayed a smooth and compact appearance. Mechanical parameters are crucial factors determining the potential application direction of investigated materials. Interestingly, the opposite results were obtained from mechanical testing; in a study by Yuan et al. [25], an increase in the tensile strength and a decrease in the elongation at break were observed.

According to research performed by Jia et al. [42], shellac improved the thermal and mechanical properties of zein films produced by coaxial electrospinning. Similarly, composites made of zein and shellac were characterized by a smooth, homogeneous morphology without any beads. The improved mechanical properties of composite films were ascribed to a uniform integration of shellac into the zein matrix. The values of the elongation at break parameters increased for shellac-modified samples of the zein composite; a similar tendency was observed in our research. However, in comparison to zein-based materials in our chitosan-based films, an increase in tensile strength was not observed. The thermal stability of the shellac/zein materials was improved, and more energy was required to disrupt the intermolecular interactions created between biopolymers. Based on FTIR spectra registered for shellac-modified samples, Jia et al. [42] noticed alterations in amide I bands to higher wavenumbers. In our samples, this shift also occurred, but we could suspect that it was related to the glycerol addition in the chitosan matrix and could not be ascribed to the presence of shellac. The same situation occurred with the amide II band position, where Jai et al. [42] observed only a decrease in the intensity, with no alteration in this absorption peak. However, a characteristic peak of shellac at about 1700 cm^−1^ was also found in the spectra.

Shellac improved the thermal stability of other polysaccharide materials such as Konjac glucomannan-based films, which were fabricated by Du et al. [43] with the intention to develop food packaging as an alternative to synthetic ones. SEM images confirmed that biopolymer blends exhibited homogeneous and continuous morphology, and no phase separation appeared, which was in line with our observations. However, Konjac glucomannan and shellac blends displayed different morphologies depending on the shellac concentrations. With an increasing shellac concentration, a structure with cracks transformed to a dense and continuous structure without cracks and pores, confirming shellac homogeneous distribution and the high miscibility of these two biopolymers, while in our films, such dependency was not observed. Furthermore, the shellac addition enhanced the mechanical properties, such as tensile strength and elongation at break. The latter results corresponded with our findings, and it could be concluded that the films were more stretched and flexible. The FTIR spectra of films containing a smaller amount of shellac were similar to the Konjac glucomannan spectrum, while higher concentrations of shellac in the films were reflected in characteristic peaks at 2930 cm^−1^. This corresponds with our findings, where no additional peaks were observed following shellac incorporation. Shifts in the absorption peaks at around 3400 cm^−1^ related to -OH functional groups confirmed that new intermolecular hydrogen bonds formed between selected biopolymer chains, which resulted in improved thermal and mechanical properties. The alteration of this band position was in contrast to our study, as well as the fact that the addition of shellac resulted in thicker films provided by the enhanced stiffness of polymer chains due to shellac and Konjac glucomannan interactions [43].

Based on FTIR analysis performed by Song et al. [26] for chitosan and shellac emulsions, the characteristic bands of the latter biopolymer after blending were no longer observed, confirming the combination of these two molecules. It was speculated that this might be related to the formation of electrostatic interactions between chitosan and shellac as well as the creation of hydrogen bonds [26].

The thermal degradation process for chitosan–shellac biocomposite fabric also occurred in two phases and, as in other mentioned research, shellac also improved the thermal stability of the investigated materials [27]. TGA analysis was also performed for adhesives consisting of shellac and chitosan. The combination of these two biopolymers caused less intense degradation, which occurred at higher temperatures than for pure shellac [23]. However, Ma et al. [29] indicated that shellac hydrogel microparticles, in addition to the chitosan hybrid film, did not affect material thermal stability.

Based on the results presented by Pinto et al. [44], glycerol influenced mechanical properties, leading to higher flexibility and lower tensile strength of chitosan films similar to the sample CS_GLY. As shellac is characterized by deteriorated mechanical strength in comparison to other natural polymers, its addition caused a decrease in the tensile strength of the chitosan films in our investigation. However, plasticizers such as glycerol or PEG 400, according to Wang et al.’s [30] experiment, might form hydrogen bonds with shellac molecules and, as a result, strengthen the film’s mechanical properties. But it can also be predicted that the decreased tensile strength in our films after the addition of shellac and glycerol might result from the discontinuous microstructure, leading to a lower mechanical strength of the materials. This might be related to the disruption of the chitosan network and the greater mobility and flexibility of polymer chains, resulting in the greater flexibility of films [45,46].

AFM images were registered for the shellac-based film developed for wound dressing applications, which indicated minor fluctuations, while the incorporation of an additive caused an uneven surface [47]. Roughness parameters were also calculated for shellac thin films. It was reported that shellac films were smooth with low roughness (root mean square) parameter values, which contributed to the creation of a uniform and smooth interface with the semiconducting polymers [48,49]. Shellac films obtained with ethanol by Irimia-Vladu et al. [50] exhibited smooth surfaces with roughness (root mean square) parameter values of about 1 nm [49]. While, based on the SEM images of the biocomposite fabric coated with chitosan and shellac, it can be concluded that the shellac addition resulted in a rougher surface of the layered structure of chitosan, and the compatibility of chitosan and shellac was confirmed [27]. Song et al. [26], in the SEM images for chitosan coating, observed cracks, while the shellac addition enhanced the toughness of the coating by linking chitosan chains, reducing clearance, and promoting molecular fluidity. Glycerol, as a plasticizer, is responsible for a more homogeneous, smooth surface and lower roughness (root mean square) of chitosan films [44,51,52], which corresponds with our conclusions. Moreover, glycerol improves the miscibility of polymer blends as, for example, chitosan and poly(vinyl alcohol) blends [52].

The water contact angle was measured for the chitosan–shellac coating used for paper and fabric; in both applications, the shellac addition led to higher values in the water contact angle [27]. The same observation was made by Ma et al. [29] for shellac hydrogel/chitosan hybrid film and by Wang et al. [30] for PVP/ shellac and PVP/carboxymethyl chitosan/copper oxide/shellac composite films. These conclusions were in opposition to the results of our study, however, our measurements were not performed for water but for glycerol. According to Soradech et al.’s [53] results, the contact angle for the shellac film measured with diiodomethane was about 62°.

Zhu et al. [35] indicated that the addition of shellac to the chitosan/sodium alginate particles caused a decrease in the swelling degree and improved gel particle stability, which was in agreement with our findings.

These observed discrepancies in the physicochemical properties of biopolymer-based materials enriched with shellac or glycerol might result from the complex composition, the specific characteristics of the applied biopolymers, and the type of interactions formed between each component. Chitosan’s characteristic properties are also crucial factors that affect modified films’ mechanical properties. Polymer-based films can be fabricated as a potential material for cutaneous application, for instance, by supporting or promoting the wound healing process. Proper mechanical resistance and the flexibility of films are essential for effective skin adhesion. The surface properties are another important feature of materials intended for biomedical purposes; the hydrophilic surface of the material can facilitate wound management, and a rougher surface might also be expected. The assessment of the behavior of the biopolymer-based films in the solutions, particularly in phosphate-buffered saline, is a basic step in characterizing the potential multifunctional biomaterials. Moreover, materials with appropriate swelling capacity could provide the proper management of wound exudate, and homogeneous and uniform blends might also promote therapeutic effects. Furthermore, the thermal stability of biomaterials should also be monitored, for example, due to sterilization treatment, to observe their integrity. Incorporated additives influence not only the physicochemical properties but also the biological activity of the produced materials for specific applications. Therefore, further research, especially biological evaluation, is necessary to assess the suitability of the obtained chitosan-based films for biomedical, cosmetic, or food packaging purposes.

## 5. Conclusions

From the conducted research, certain conclusions can be drawn. Homogeneous and smooth chitosan films enriched with selected concentrations of shellac and glycerol as a plasticizer were obtained, and all conducted experiments confirmed that glycerol and shellac influenced the physicochemical properties of the chitosan film. The effect was observed particularly on the roughness parameters, contact angle, surface free energy, mechanical properties (Young’s Modulus, tensile strength, and elongation at break), and swelling degree. FTIR spectra exhibited no significant alterations in the wavenumbers of the characteristic bands of chitosan after modification. Modified films were characterized by slightly lower surface roughness, which corresponded with surface topography from AFM images and enhanced surface free energy in comparison to the pure chitosan sample. For higher concentrations of shellac in the samples, a decrease in the contact angle measured for the hydrophilic liquid was observed; thus, the hydrophilicity of the film surface increased. The TG and DTG thermograms for all samples were similar; however, in the range from 90 °C to 180 °C, a decreased weight loss occurred in shellac-incorporated films. These types of samples also presented a lower Young’s Modulus, lower tensile strength, and higher elongation at break in comparison to chitosan film, and the most flexible was a film with the highest amount of shellac. At a pH of 7.4, an almost five times lower swelling degree was noticed for glycerol/shellac-modified samples and a slower disintegration process than for pure chitosan. Shellac and glycerol can effectively modify the physicochemical properties of chitosan films. In terms of biomedical applications, for instance, in the development of wound dressing materials, a modification toward increased surface hydrophilicity, surface roughness, film flexibility, proper swelling ability, and thermal stability, which were observed particularly for chitosan films enriched with glycerol and larger amounts of shellac, may support the proper wound management or wound healing process. Furthermore, due to the non-toxicity, biocompatibility, and biodegradability of both biopolymers, they may act as a matrix for incorporating active substances and find applications as food packaging, biomaterials, drug delivery systems, or in cosmetics and pharmaceuticals.

## Figures and Tables

**Table 1:** Composition of chitosan-based films.

Specimen	2% Chitosan Solution in 0.1 mol/L Acetic Acid Solution [g]	5% Shellac Solution in Ethyl Alcohol [g]	Glycerol [g]
CS	25	-	-
CS_GLY	25	-	0.25
CS_GLY_SH 0.5%	25	0.05	0.25
CS_GLY_SH 1%	25	0.1	0.25
CS_GLY_SH 2%	25	0.2	0.25
CS_GLY_SH 2.5%	25	0.25	0.25

**Table 2:** Wavenumbers of characteristic bands for chitosan-based films.

Specimen	Characteristic Bands [cm^−1^]							
	-OH and -NH Stretch	C-H Stretch	Amide I	Amide II	CH_2_ Bend	Amide III	C-O Stretch/C-O-C Asymmetric Stretch	C-O Stretch/C-O-C Symmetric Stretch
CS	3356	2928/2879	1637	1557	1408	1259	1072	1029
CS_GLY	3357	2926/2881	1651	1564	1413	1257	1075	1040
CS_GLY_SH 0.5%	3360	2931/2881	1651	1564	1413	1257	1075	1042
CS_GLY_SH 1%	3357	2928/2881	1647	1564	1413	1256	1075	1041
CS_GLY_SH 2%	3357	2930/2881	1651	1564	1416	1257	1075	1041
CS_GLY_SH 2.5%	3358	2929/2880	1651	1564	1416	1255	1074	1042

**Table 3:** Rq and Ra values for chitosan films with glycerol and shellac addition (scanned area of 5 μm × 5 μm).

Specimen	Rq [nm]	Ra [nm]
CS	7.37 ± 0.82	5.88 ± 0.71
CS_GLY	2.69 ± 0.57	2.07 ± 0.44
CS_GLY_SH 0.5%	1.78 ± 0.03	1.41 ± 0.02
CS_GLY_SH 1%	3.17 ± 0.03	2.52 ± 0.05
CS_GLY_SH 2%	4.22 ± 0.16	3.35 ± 0.13
CS_GLY_SH 2.5%	3.74 ± 0.60	2.81 ± 0.40

**Table 4:** Contact angle (glycerol and diiodomethane) and surface free energy results for chitosan-based films.

Specimen	Θ^G^	Θ^D^	γ_s_ [mJ/m^2^]	γ_s_^d^ [mJ/m^2^]	γ_s_^p^ [mJ/m^2^]
CS	92.9 ± 4.3	54.0 ± 2.7	31.99	31.63	0.37
CS_GLY	89.6 ± 5.6	50.8 ± 5.5	33.58	32.94	0.64
CS_GLY_SH 0.5%	90.9 ± 2.4	38.9 ± 4.0	41.11	41.06	0.04
CS_GLY_SH 1%	93.7 ± 5.6	48.2 ± 3.9	35.93	38.85	0.07
CS_GLY_SH 2%	85.3 ± 5.4	44.4 ± 4.1	36.91	35.95	0.96
CS_GLY_SH 2.5%	80.7 ± 3.3	44.6 ± 3.3	36.64	34.53	2.11

**Table 5:** Parameters of TG and DTG curves obtained for chitosan-based films.

	Parameter	CS	CS_GLY	CS_GLY_SH 0.5%	CS_GLY_SH 1%	CS_GLY_SH 2%	CS_GLY_SH 2.5%
Step I	T_max1_ [°C]	94.1	89.9	88.4	89.2	88.7	82.9
	Mass loss [%/min]	−2.54	−2.33	−1.86	−1.91	−1.89	−1.94
	Mass loss in T_max1_ [%]	5.94	4.96	4.42	4.11	4.36	4.41
Step II	T_max2_ [°C]	295.9	291.9	292.9	294.4	292.1	293.8
	Mass loss [%/min]	−16.15	−17.88	−16.61	−15.92	−16.42	−15.75
	Mass loss in T_max2_ [%]	29.98	32.14	30.61	30.13	32.14	33.20
	Residual mass [%]	39.46	36.22	37.17	37.4	35.42	35.32

## Data Availability

The original contributions presented in this study are included in the article. Further inquiries can be directed to the corresponding author.
